# Low temperature difference thermoacoustic prime mover with asymmetric multi-stage loop configuration

**DOI:** 10.1038/s41598-017-08124-5

**Published:** 2017-08-09

**Authors:** T. Jin, R. Yang, Y. Wang, Y. Feng, K. Tang

**Affiliations:** Institute of Refrigeration and Cryogenics/Key Laboratory of Refrigeration and Cryogenic Technology of Zhejiang Province, Zhejiang University, Hangzhou, 310027 China

## Abstract

Environmentally friendly and low-cost technologies to recover low-grade heat source into usable energy can contribute to ease the energy shortage. Thermoacoustic technology is expected as one promising approach in this ascendant field. In this work, the multi-stage looped thermoacoustic prime movers with asymmetric configuration, which can provide travelling-wave resonator and appropriate acoustic field for efficient regenerator, have been proposed and experimentally studied. The presented looped thermoacoustic prime movers can start to oscillate with quite low temperature difference along the regenerator. The lowest onset temperature difference obtained in the experiments is only 17 °C (the corresponding heating temperature is 29 °C), which can be achieved in both three-stage and four-stage looped thermoacoustic prime movers, with CO_2_ of 1 MPa or 1.5 MPa as the working fluid. An electric generator driven by a three-stage looped thermoacoustic prime mover with low heating temperature was tested to achieve the acoustic to electric conversion.

## Introduction

Low-grade thermal energy, such as solar thermal energy, geothermal energy, ocean thermal energy and waste heat, is enormously reserved but not yet effectively utilized^1–3^. For instance, in 2015, around 6.2 × 10^19^ J energy was wasted as water effluent, steam and air, corresponding to 61% of the total energy consumption (including electricity generation, residential consumption, commercial consumption, industrial process, and transportation) in the United States^3^. In the past few decades, some technologies have been developed for recovering the low-grade heat sources, e.g., to generate electricity by organic Rankine cycle, to produce refrigeration by absorption/adsorption cooling cycle^4, 5^. Thermoacoustic engine, an emerging technology for converting thermal energy into acoustic power or vise versa, is also expected as a promising technology of utilizing these thermal energy^6^. Thanks to the lack of moving part, the thermoacoustic engines possess the merits of high reliability and environmental friendliness and have demonstrated promising prospects in electric generation, refrigeration, gas separation and water pumping^6–8^. However, a traditional thermoacoustic prime mover generally requires high-temperature heat source to achieve effective acoustic power output, which is a huge barrier to utilizing the low-grade heat source. To reduce the onset and operating temperatures of a thermoacoustic engine is thus of great importance for its application in the field of low-grade heat source recovery.

A looped travelling-wave thermoacoustic engine can theoretically execute a reversible thermodynamic cycle analogous to that in a Stirling engine. However, it was ever troubled with low acoustic impedance in the regenerator, which can cause severe viscous loss, when it was firstly proposed^9, 10^. The thermoacoustic Stirling heat engine invented by Backhaus and Swift successfully solved this problem by proper combination of a loop structure and a standing-wave resonator^11^, which could achieve the thermal efficiency as high as 30%. However, the required heating temperature was generally above 600 °C, mainly due to the severe viscous dissipation in its standing-wave resonator^6^. De Blok modified the looped travelling-wave thermoacoustic engine by inserting multiple thermoacoustic cores into the loop^12^, which significantly lowered the required temperature of heat source, and attained a relative Carnot efficiency of 38% when driven by the heat source of 99 °C. Bi *et al*. proposed a three-stage looped thermoacoustic electric generator^13^, which achieved the maximum electric power of 4.69 kW with thermal-to-electric efficiency of 15.6% and the maximum thermal-to-electric efficiency of 18.4% with electric power of 3.46 kW, when the heating temperature was 650 °C. However, the existing looped thermoacoustic systems generally depend on their symmetric configuration to ensure the regenerator to be located at the position where the travelling-wave component dominates. As a result, the configuration of the looped thermoacoustic system is subjected to many constraints, i.e., the requirement of symmetric configuration (including the coupling with loads) will limit its structural form and increase the complexity in manufacture^12, 13^.

In the current work, various asymmetric looped thermoacoustic prime movers (LTAPMs), including one-stage, two-stage, three-stage and four-stage LTAPMs, aiming to be driven by low-grade thermal energy will be proposed. Experiments have then been carried out to validate the design, in which efforts are especially focused on the operating features such as onset temperature, pressure ratio and heating temperature. A three-stage LTAPM has also been used to drive a linear alternator, as an example, for preliminarily verifying its applicability to electric generation with low-grade heat source.

## Results

**Figure 1:**
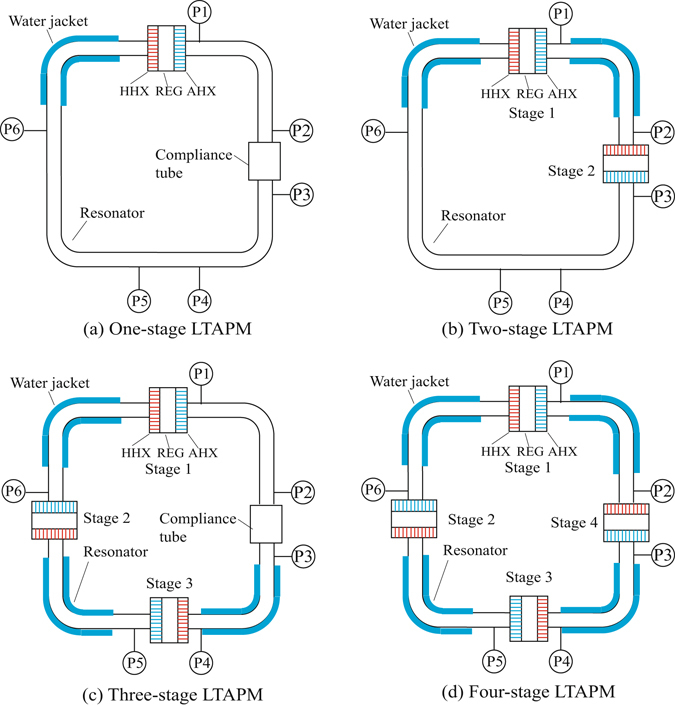
Schematic of four typical LTAPMs.

Four typical LTAPMs (including one-stage, two-stage, three-stage and four-stage systems) have been proposed, designed and experimented, as shown in Fig. 1. The dimensions of the LTAPMs can be found in Table 1 in the “Methods” section. For the one-stage and three-stage LTAPMs, a compliance tube has been adopted to adjust the acoustic field. The four-stage LTAPM is similar to that in Ref. 12, which can be regarded as one of typical cases in our proposal.

**Table 1:** Dimensions of the main parts of LTAPMs.

Items	HHX	REG	CHX	Resonator	Compliance tube
Length/mm	30	30	30	—	90
Diameter/mm	99	99	99	26	72
Volumetric porosity	0.26	0.74	0.26	1	1
Hydraulic radius/mm	0.5	0.05	0.4	6.5	18

### Acoustic field in the LTAPMs

**Figure 2:**
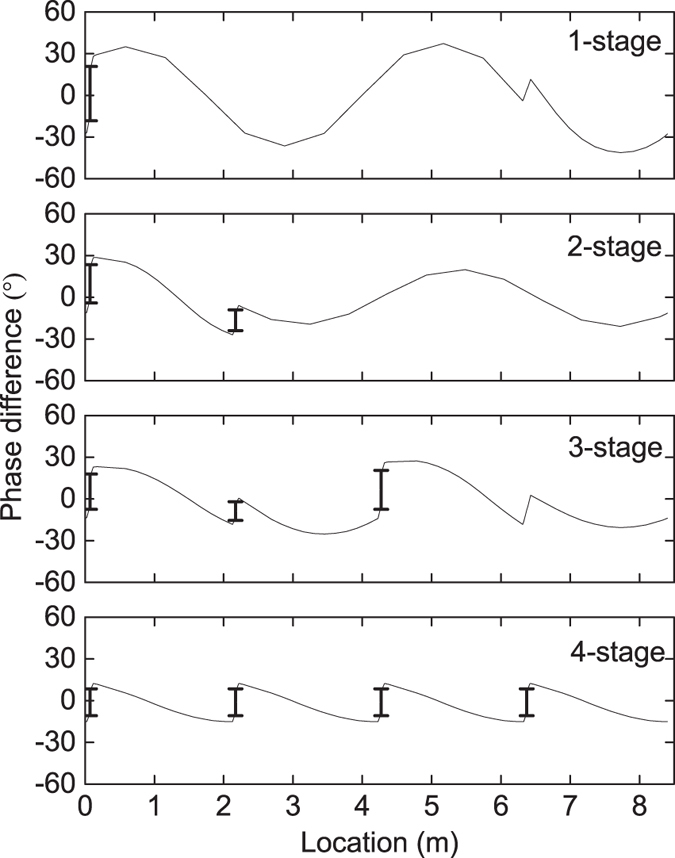
Distribution of phase difference in four LTAPMs (the range marked by heavy line segment representing the regenerator).

Simulation has been firstly conducted to verify whether appropriate acoustic field can be established or not in these four LTAPMs, with the help of DeltaEC (Design Environment for Low-amplitude Thermoacoustic Energy Conversion)^14^. Figure 2 shows the distribution of phase difference (defined as the phase angle of *p*_1_ minus that of *U*_1_) in four systems. As can be seen, in the one-stage LTAPM, the phase difference of the regenerator is between −18° and 21°. In the two-stage LTAPM, the phase difference of the two regenerators ranges from −4° to 23° and from −24° to −9°, respectively. In ﻿the﻿ three-stage LTAPM, the phase difference of the three regenerators ranges from −7° to 18°, from −15° to −2° and from −7° to 21°, respectively. In the four-stage LTAPM, the phase difference of each regenerator ranges from −11° to 8°. The results indicate that all the regenerators in four configurations have been located near the pure travelling-wave positions. The travelling wave field is one of the key issues in lowering the onset temperature and the operating temperature, since the acoustic field dominated by the travelling-wave field can enhance thermoacoustic conversion in the regenerator (see Equation (3) in the “Discussion” section). In brief, the appropriate acoustic field in the systems can be built by the proper arrangement of the regenerators and the introduction of the compliance tube.

### Operating features of the LTAPMs without load

**Figure 3:**
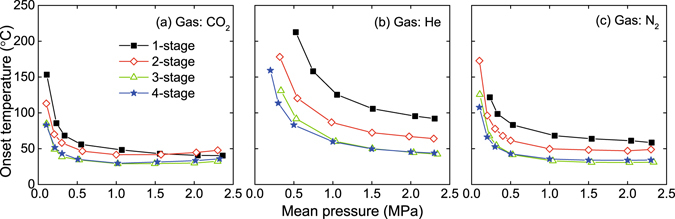
Onset temperatures under different mean pressures.

Systematic experiments have been conducted on the physical devices to study the performance of the LTAPMs. Carbon dioxide (CO_2_), helium (He) and nitrogen (N_2_) were adopted as working fluids, respectively. Figure 3 displays the onset temperatures under different mean pressures. When the mean pressure is above 1 MPa, most of the LTAPMs can start to oscillate with a heating temperature below 100 °C, indicating that the LTAPMs possess the superiority in harvesting low-grade heat. When the working fluid is CO_2_, all the four LTAPMs can start to oscillate with a heating temperature below 60 °C when the mean pressure is above 0.5 MPa. When the mean pressure is below 0.5 MPa, the onset temperature is also still very low, which helps to reduce the requirements in pressure resistance of the system. For instance, when the CO_2_ of 0.1 MPa is adopted as working fluid, the three-stage and four-stage LTAPMs can start to oscillate at the heating temperatures below 90 °C. Furthermore, the onset temperature can be effectively lowered when the quantity of regenerators is increased to three or four. The lowest onset temperature is only 29 °C (with a temperature at the cold end is 12 °C, and when the CO_2_ of 1 MPa or 1.5 MPa is adopted).

**Figure 4:**
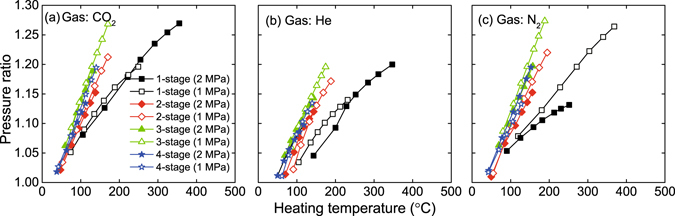
Pressure ratio versus heating temperature.

Figure 4 presents the pressure ratio (defined as the ratio of maximum over minimum of the pressure oscillation) versus the heating temperature. It is obvious that the pressure ratio rises with the elevated heating temperature. The volumetric velocity flows out of the regenerator is roughly proportional to the ratio of the heating temperature over the temperature at the cold end^11^. In the experiments, the variation in the temperature at the cold end is very small compared with that in the heating temperature. Thus, the amplification of acoustic power increases with the elevated heating temperature and the pressure ratio, which reflects the intensity of oscillation, also rises. To achieve a certain pressure ratio, the corresponding heating temperature can be reduced by increasing the quantity of regenerators when it is less than three. However, to achieve the given pressure ratio, the corresponding heating temperature of the four-stage LTAPM is even higher than that of the three-stage LTAPM. In the experiments, the highest pressure ratio is 1.27, when the CO_2_ or N_2_ of 1 MPa is adopted and the heating temperature is 170 °C or 188 °C. The results show that the LTAPM can achieve a considerably high pressure ratio with a heating temperature much lower than that of a Stirling thermoacoustic engine, whose required heating temperature is typically above 600 °C to achieve a similar pressure ratio^6^.

### Applicability verification for electric generation

**Figure 5:**
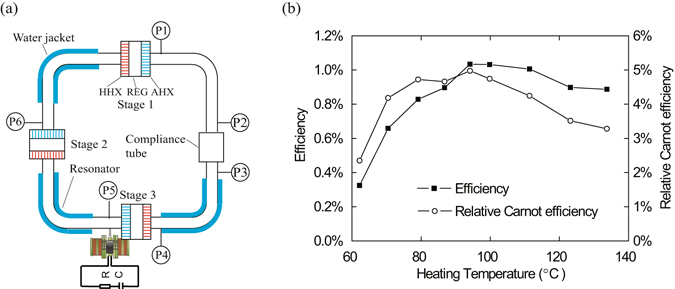
Schematic of the electric generator driven by a three-stage LTAPM and its efficiency and relative Carnot efficiency versus heating temperature.

To demonstrate the potential applicability of the LTAPM with low-grade heat source, an electric generator driven by a three-stage LTAPM, as shown in Fig. 5(a), has been preliminarily studied. A variable electric R-C load is connected to the linear alternator to output electricity. The working fluid is the He-CO_2_ gas mixture, of which 90% is helium, and the other 10% is CO_2_, which helps to adjust the frequency to be closer to the mechanical resonant frequency of linear alternator. Figure 5(b) shows the efficiency and the relative Carnot efficiency at different heating temperatures when the mean pressure is 2 MPa. When the heating temperature is 94 °C, the thermal-to-electric efficiency reaches its maximum value 1.03%, corresponding to 5% of Carnot efficiency, with the R-C load of 11 Ω and 141 μF. It should be noted that the conversion from acoustic power to electricity in the present linear alternator is yet far from highly efficient, considering that it was directly modified from an available linear compressor by reversing its functioning direction. Further optimization that is of great necessity has been scheduled in our near future efforts.

## Discussion

In order to design the LTAPMs that are not restricted to symmetric configuration for being efficiently powered by low-grade heat source, efforts should be focused on the following two issues.

(1) Arrangement of regenerators. The onset temperature, a key issue for the use of low-grade heat source, can be lowered by increasing the quantity of regenerators^15^. The onset process can be discussed in terms of the quality factor *Q* of the LTAPM^16^, which is defined as

1 $$Q=-\omega {E}_{{\rm{st}}}/W,$$

where *ω* is the angular frequency, *E*_st_ is the acoustic power stored in the LTAPM, *W* is the acoustic power generated or consumed. Imagine a process in which the thermoacoustic prime mover is forced to oscillate, while the heating temperature rises steadily from the temperature at the cold end. At the beginning, all components in the thermoacoustic prime mover (including heat exchangers, regenerators and resonator) consume acoustic power, thus *W* has a negative value. As the heating temperature continues to rise, the acoustic power generated in the regenerator will be increased, thus *W* begins to rise towards zero. When the heating temperature reaches a critical value, the acoustic power generated in the regenerator balances the acoustic power dissipated in the thermoacoustic prime mover, and *W* turns to zero. At this moment, *Q* is infinite, indicating that the system is in an unstable state. As a result, any further rise in heating temperature will lead to the oscillation onset in the thermoacoustic prime mover. Hence, the heating temperature with which *W* turns to zero is called the onset temperature.

In the following analysis, a LTAPM will be simplified as a combination of the regenerator(s) with temperature gradient and the connecting resonator segments. When the heating temperature reaches the onset temperature,

2 $$W={W}_{{\rm{res}}}+\sum _{n=1}^{N}{W}_{{\rm{reg}}}=0,$$

where *N* is the quantity of regenerators, *W*_res_ is the acoustic power consumed in the resonator, and *W*_reg_ is the acoustic power generated in a regenerator. As the quantity of regenerators is increased, the dissipation needing to be overcome by each regenerator becomes less. There exists a positive correlation between the acoustic power gain by the thermoacoustic conversion and the temperature difference in the regenerator^11^. Hence, when the quantity of regenerators is increased, the onset temperature will be lowered. Furthermore, the increased quantity of regenerator(s) may also enhance the thermal efficiency^17^. Hence, an analysis on the performance of the system with various quantities of regenerators is of significance for optimization.

Another factor that influences the performance of the thermoacoustic engine is the position of the regenerators. According to the thermoacoustic theory^18^, the variation in the acoustic power through an element channel of the regenerator with the length of d*x* in one dimensional case can be depicted as

3 $$\frac{{\rm{d}}{E}_{2}}{{\rm{d}}x}=-\frac{{r}_{{\rm{\nu }}}}{2}{|{U}_{1}|}^{2}-\frac{1}{2{r}_{{\rm{\kappa }}}}{|{p}_{{\rm{1}}}|}^{{\rm{2}}}+\frac{1}{2{T}_{{\rm{m}}}}\frac{d{T}_{{\rm{m}}}}{dx}|{p}_{{\rm{1}}}||{U}_{{\rm{1}}}|\cos \,\phi $$

where *E*_2_ is the acoustic power, *r*_ν_ is the resistance caused by viscosity, *r*_κ_ is the resistance caused by thermal relaxation, *T*_m_ is the mean temperature, *U*_1_ and *p*_1_ stand for the volumetric velocity and pressure, respectively, and *φ* presents the phase difference between *U*_1_ and *p*_1_. Equation (3) indicates that the regenerator should be located at the position where the phase difference *φ* is close to zero, in order to execute a reversible cycle. This is significant for the attainment of both low onset temperature and high thermal efficiency. If the regenerator is installed in an inappropriate position, the phase difference will deviate from zero, and the increase in the quantity of regenerators may not definitely lower the onset temperature.

In a looped thermoacoustic engine, there exists standing-wave component because of the reflection of acoustic wave from the locally enlarged thermoacoustic core. One of the typical distributions of the standing-wave component at fundamental frequency in the looped configuration contains two peaks and two troughs for pressure amplitude *p*_1_ and volumetric velocity amplitude *U*_1_, which can be verified by the simulation of looped thermoacoustic systems with asymmetric configuration in Refs. 19–21. The phase differences at the peak and the trough of pressure amplitude (or the trough and the peak of volumetric velocity amplitude) are zero. Thus, there will be four pure travelling-wave points in the loop where the phase differences are zero, and the distance between the adjacent points is roughly 1/4 wavelength. Hence, these pure travelling-wave points are considered as the appropriate positions for installing the regenerator, based on which the LTAPMs in this work were proposed.

Additionally, it should also be noticed that the multi-stage thermoacoustic systems with symmetric configurations, for instance, the three-stage thermoacoustic electric generator in Ref. 13, can also run effectively. These symmetrically arranged systems consist of several identical thermoacoustic cores with equivalent distance, thus the distribution of pressure amplitude and volumetric velocity amplitude in each segment (including the thermoacoustic core and the connecting resonator) will be the same. By properly designing the locally enlarged thermoacoustic core and the resonator, it is feasible to achieve an acoustic field dominated by travelling wave in the regenerators.

(2) Phase adjustment. To install the regenerators at or near the positions where the phase difference is close to zero, proper phase adjustment is needed. Ascribed to the fact that there is no soft or hard boundary in the looped resonator and the components of the thermoacoustic engine are highly coupled with each other, the acoustic field is very sensitive to the variation in acoustic impedance, which brings difficulty to the fine-tuning of acoustic field. Thus, the sensitivity should firstly be reduced. De Blok’s system reduced this sensitivity by adopting the symmetric configuration^12^. When this sensitivity is reduced, the acoustic field can be further adjusted by changing the diameter of resonator. It is well known that the variation in the phase angle of pressure *ph*(*p*_1_) can be accelerated by reducing the cross-sectional area of the channel, while the variation in the phase angle of volumetric velocity *ph*(*U*_1_) should be accelerated by expanding it.

In our experiments, a compliance tube (a locally enlarged tube, as shown in Fig. 1) is used to actively adjust the acoustic field in one-stage and three-stage LTAPMs. By inserting a compliance tube in the resonator, a boundary similar to the soft boundary can be created. Accordingly, the spatial distribution of the acoustic field can be roughly determined, and the expected position for the regenerator (generally the peak of *p*_1_) can be identified, laying foundation for the fine-tuning of the acoustic field. Besides, the variation in the phase angle of volumetric velocity *ph*(*U*_1_) is accelerated while the variation in the phase angle of pressure *ph*(*p*_1_) is decelerated, leading to the accordingly altered phase difference. In the LTAMPs, the role of each thermoacoustic core (including the regenerator, the hot heat exchanger and the ambient heat exchanger) is not only the thermoacoustic conversion, but also the phase adjustment. The cross-sectional area of the thermoacoustic core is much larger than the resonator, thus the variation of *ph*(*U*_1_) is accelerated while the variation of *ph*(*p*_1_) is decelerated, which is similar to the function of a compliance tube. As discussed in the “Results” section, the four-stage LTAPM demonstrates good performance and appropriate acoustic field. If one of the thermoacoustic cores is replaced by a compliance tube which provides equivalent variation in *ph*(*U*_1_) and *ph*(*p*_1_), then the other three thermoacoustic cores can also work effectively. Based on this assumption, the dimensions and installation position of the compliance tube in the three-stage LTAPM can be roughly determined. Similarly, the two-stage LTAMP can be used as the reference for determining the dimensions and installation position of the compliance tube in the one-stage LTAMP.

In addition, proper design of acoustic field can also reduce the acoustic dissipation in the resonator. The acoustic power propagating in the resonator can be expressed as 0.5|*p*_1_||*U*_1_|cos*φ*. If the acoustic wave in the resonator deviates from the travelling wave, cos*φ* will be less than one, requiring larger |*p*_1_||*U*_1_| to deliver the same amount of acoustic power. The larger |*p*_1_||*U*_1_| will then induce more severe dissipation since the dissipation is proportional to |*U*_1_|^2^ and |*p*_1_|^2^ (according to Equation (3)). Thus, the acoustic field in the resonator should also be near travelling wave.

As mentioned in the “Results” section, when the quantity of regenerators is less than three, the increased quantity will cause a dramatic decrease of the onset temperature and the required heating temperature at a given pressure ratio. However, the four-stage LTAPM does not possess obvious advantages over the three-stage LTAPM, which can be attributed to the following factors. Firstly, according to Equation (2), the effect of lowering the onset temperature by adding stages will gradually diminish as the stages is increased. Hence, the drop of onset temperature, when the quantity of regenerators is increased from three to four, is less than that when the quantity is increased from one to two or from two to three. Secondly, as the quantity of regenerators is increased, the quantity of HHXs should also be correspondingly increased, which will deliver more heat into the resonator. Due to the performance limitation from the water jacket, the spatial average temperature of the resonator will be elevated, which can lead to higher viscosity, and thus more acoustic dissipation.

A traditional thermoacoustic prime mover generally requires high-temperature heat source to realize effective acoustic power output, which has inhibited its application to utilizing the low-grade heat source. In this work, on the basis of the discussion with regard to two pertinent issues, namely the arrangement of the regenerators and the phase adjustment in the loop structure, the LTAPMs with multiple stages that aimed at low temperature operation were designed. Experimental results show that all the LTAPMs can be initiated to oscillate under a relatively low heating temperature. The lowest onset temperature was only 29 °C, when the temperature at the cold end is 12 °C and the CO_2_ of 1 MPa or 1.5 MPa is adopted as the working fluid. An electric generator driven by a three-stage LTAPM was also tested to realize the electricity output. In summary, the presented LTAPMs can provide a potential approach to recovering low-grade heat source.

## Methods

### Details of the experimental set-up

**Figure 6:**
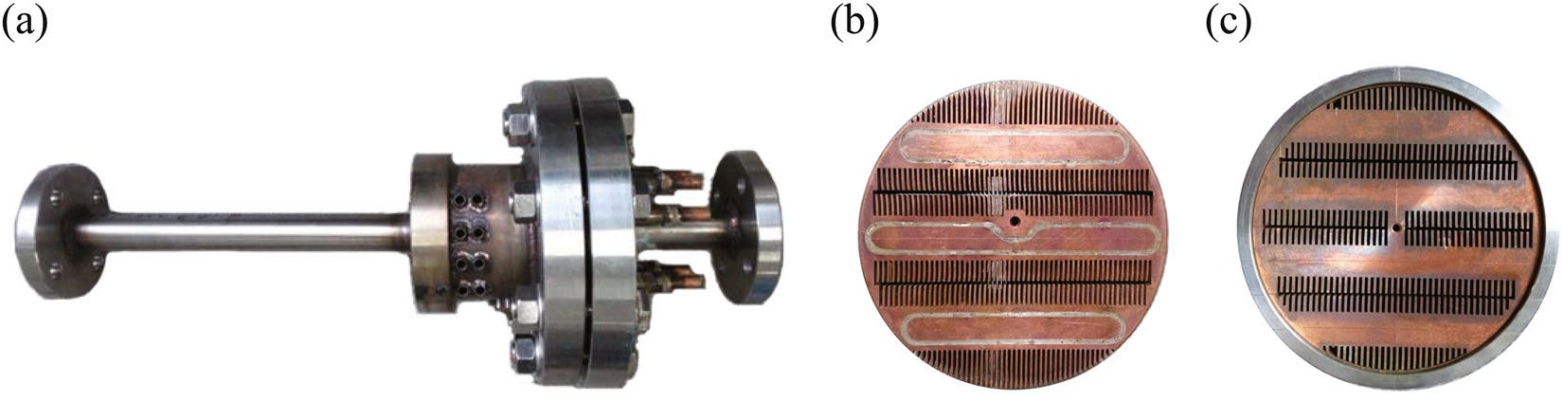
Photos of main components: (**a**) thermoacoustic stage, (**b**) ambient heat exchanger, (**c**) hot heat exchanger.

The distances between adjacent regenerators in two-stage, three-stage and four-stage LTAPMs, or between the regenerator and the adjacent compliance tube in one-stage and three-stage LTAPMs are approximately 1/4 wavelength. To achieve high acoustic impedance, the cross-sectional area of the thermoacoustic core (including a hot heat exchanger, a regenerator and an ambient heat exchanger) has been locally enlarged. Dimensions of the main parts are listed in Table 1. The total loop lengths of the four LTAPMs are all 8.2 m. Figure 6(a) shows the photo of thermoacoustic stage, in which the regenerator is a stack of 120 mesh stainless-steel screens. The ambient heat exchanger is a kind of fin-type heat exchanger as shown in Fig. 6(b). Both the fin width and the spacing between fins are 0.8 mm. Water flows inside the copper block to carry away the rejected heat. The configuration of the hot heat exchanger is similar to the ambient heat exchanger, as shown in Fig. 6(c). Both the fin width and the spacing between fins are 1 mm. 12 cartridge heaters are inserted into it to input heat.

The linear alternator was directly modified from an available pressure wave generator (2s102W, Qdrive) by reversing its functioning direction. It consists of two resonant linear motors, with clearance seal pistons, working into a common chamber, ported to a load interface, and all enclosed in a bolted pressure vessel. The parameters of the linear alternator are listed in Table 2.

**Table 2:** Parameters of the linear alternator.

Items	Value
Mechanical resistance/(N·s/m)	4.65
Mechanical stiffness/(N/m)	34086
Moving mass/kg	0.51
Inductance/mH	83.9
Electrical resistance/Ω	6.93
Transduction coefficient/(N/A)	49.4
Piston area/m^2^	0.00139
Back volume/m^3^	0.00049

### Details of the simulation

DeltaEC is a freely available and widely used software from the Los Alamos National Laboratory for analyzing one‐dimensional acoustical networks^11, 14, 18^. The parameters of the simulation model are the same with those of the experimental set-up, as shown in Table 1. The HX segment was chosen to calculate the heat exchangers. The values of *GasA/A* and *y*_0_ in the segment are the same as the volumetric porosity and the hydraulic radius of the corresponding heat exchanger. The STKSCREEN segment was adopted to calculate the regenerator, where *VolPor* and *r*_h_ are the volumetric porosity and the hydraulic radius of the physical regenerator. The model in the present work simulates the free oscillation in the thermoacoustic engine driven by the heat above onset temperature. The working fluid is the helium of 2 MPa. The pressure ratios at the position of P1 in four systems were all set at 1.1. The total heating powers for one-stage, two-stage, three-stage and four-stage LTAPMs are 609 W at 143 °C, 1506 W at 98 °C, 2190 W at 88 °C, and 3623 W at 83 °C, respectively. The required heating temperature drops with the increase of stage quantities, verifying the possibility of lowering the onset and operating temperatures of the thermoacoustic engine by adopting multiple stages.

### Measurement

In the thermoacoustic core, K-type thermocouples are adopted to measure the heating temperature and the temperature at the cold end. The signals from the thermocouples are acquired by an Agilent 34970 A data acquisition switch unit with an Agilent 34901 A 20-channel multiplexor module. Six pressure sensors (GE UNIK 5000 series) are installed along the loop, whose signals are acquired by an NI 9205 analog input module. The temperature and the pressure are both measured by a time interval of 5 s, and the data are displayed and recorded by the data collection program based on LabVIEW.

### Data Availability

The datasets generated and analyzed during the current study are available from the corresponding author on reasonable request.
